# Late-Onset Antiphospholipid Syndrome Presenting With Recurrent Multifocal Ischemic Strokes: Diagnostic and Management Challenges

**DOI:** 10.7759/cureus.113913

**Published:** 2026-08-03

**Authors:** Mariam Ahmed, Mohamed A Ahmed, William Boadu

**Affiliations:** 1 Medicine, Qatar University, Doha, QAT; 2 Anatomical Sciences, St. George's University School of Medicine, St. George's, GRD; 3 Neurology, Mater Olbia Hospital, Olbia, ITA

**Keywords:** antiphospholipid antibody, antiphospholipid syndrome, hypercoagulability, ischemic stroke, thromboembolic event

## Abstract

Antiphospholipid syndrome (APS) is an autoimmune prothrombotic disorder that predominantly affects women of childbearing age and is a recognized cause of ischemic stroke. In older adults, APS is uncommon and frequently overlooked because cerebrovascular events are often attributed to traditional vascular risk factors, resulting in delayed diagnosis and treatment.

We report the case of a 65-year-old woman with a history of hypertension, dyslipidemia, chronic kidney disease, recurrent transient ischemic attacks (TIAs), previous intraventricular hemorrhage, and intracranial aneurysm repair, who presented with dysarthria, gait ataxia, and quadriparesis. She had experienced multiple recurrent cerebrovascular events over several years despite extensive vascular and cardiac investigations and appropriate antiplatelet therapy. Brain magnetic resonance imaging (MRI) demonstrated multiple acute multifocal ischemic infarcts involving both cerebral hemispheres on a background of extensive chronic ischemic changes. Laboratory evaluation revealed mild thrombocytopenia and a high-risk antiphospholipid antibody (aPL) profile, including positive lupus anticoagulant (LA) and markedly elevated anti-β2-glycoprotein I (β2GPI) IgG antibodies, while anticardiolipin (aCL) IgG and IgM antibodies were negative. As APS classification criteria require persistent aPL positivity on repeat testing at least 12 weeks apart, initiation of long-term anticoagulation was deferred pending confirmatory testing. This decision was further influenced by the patient’s increased bleeding risk due to previous intracranial hemorrhage. Aspirin therapy continued while she underwent rehabilitation and awaited repeat antibody testing.

This case highlights the diagnostic and therapeutic challenges of elderly-onset APS presenting with recurrent multifocal ischemic strokes. APS should be considered in older patients with recurrent or unexplained ischemic strokes, particularly when routine vascular and cardiac evaluations do not identify an adequate cause. Early recognition and appropriate serological testing may reduce diagnostic delays and facilitate secondary stroke prevention. Management requires individualized risk-benefit assessment, especially when anticoagulation is considered in patients with significant bleeding risk.

## Introduction

Antiphospholipid syndrome (APS) is a systemic autoimmune disorder characterized by the persistent presence of antiphospholipid antibodies (aPL) and a clinical predisposition to thrombosis and pregnancy-related morbidity. The pathogenic mechanisms include endothelial activation, platelet activation, complement-mediated inflammation, and disruption of anticoagulant pathways, resulting in a prothrombotic state [1,2]. Cerebral arterial thrombosis is a common neurological manifestation of APS, and ischemic stroke represents one of its most frequent initial clinical presentations [3]. Compared with the general population, individuals with APS have a significantly increased risk of ischemic stroke, with a pooled hazard ratio of 1.76 reported in cohort studies [4].

Despite this association, recognizing APS-related stroke can be challenging, particularly in older adults, where cerebrovascular events are frequently attributed to competing vascular risk factors such as hypertension, dyslipidemia, and atherosclerotic disease. Although late-onset APS has been increasingly reported, it may remain underrecognized because ischemic stroke in elderly patients is commonly presumed to result from conventional vascular mechanisms [5].

We report the case of a 65-year-old woman with recurrent multifocal ischemic strokes in whom the etiology of repeated cerebrovascular events remained unclear despite extensive vascular and cardiac evaluation. This case highlights the importance of considering APS in older patients with recurrent stroke, particularly when common etiologies fail to fully explain the clinical and radiological findings. It also emphasizes the therapeutic challenges of managing APS in patients with multiple comorbidities and competing thrombotic and hemorrhagic risks.

## Case presentation

A 65-year-old woman with a complex neurovascular history presented to the emergency department with a two-day history of dysarthria and gait disturbance. On the morning of admission, she awoke with dysarthria, gait ataxia, quadriparesis, and severe hypertension (systolic blood pressure >220 mmHg), which improved following intravenous labetalol administration. Neurological examination demonstrated lower-limb spasticity.

Her medical history was significant for hypertension, dyslipidemia, chronic kidney disease, recurrent transient ischemic attacks (TIAs), and previous tobacco use. She had also been hospitalized previously for a hemorrhagic event complicated by symptomatic intraventricular hemorrhage. Over several years, she experienced multiple self-limited episodes of acute vertigo without focal neurological deficits, which were initially considered nonspecific.

Two years before the current presentation, she developed transient jargon aphasia and confusion. Brain magnetic resonance imaging (MRI) demonstrated multiple fluid-attenuated inversion recovery (FLAIR) hyperintense lesions with confluent areas involving the infratentorial white matter, interpreted as chronic ischemic changes. Cerebral angiography identified an unruptured 10-mm basilar artery saccular aneurysm and a 2-mm carotid siphon aneurysm, for which endovascular coil embolization was performed. She was subsequently discharged on aspirin therapy.

Following each previous TIA, the patient received dual antiplatelet therapy (DAPT) for 21 days together with high-intensity statin therapy before transitioning to long-term aspirin. Repeated comprehensive cerebrovascular investigations, including non-contrast head computed tomography (CT), computed tomography angiography (CTA), and brain MRI, consistently demonstrated no large-vessel occlusion or hemodynamically significant intracranial or extracranial stenosis. Extensive cardiac evaluation, including transthoracic and transesophageal echocardiography and prolonged cardiac rhythm monitoring, was repeatedly unremarkable.

**Figure 1:**
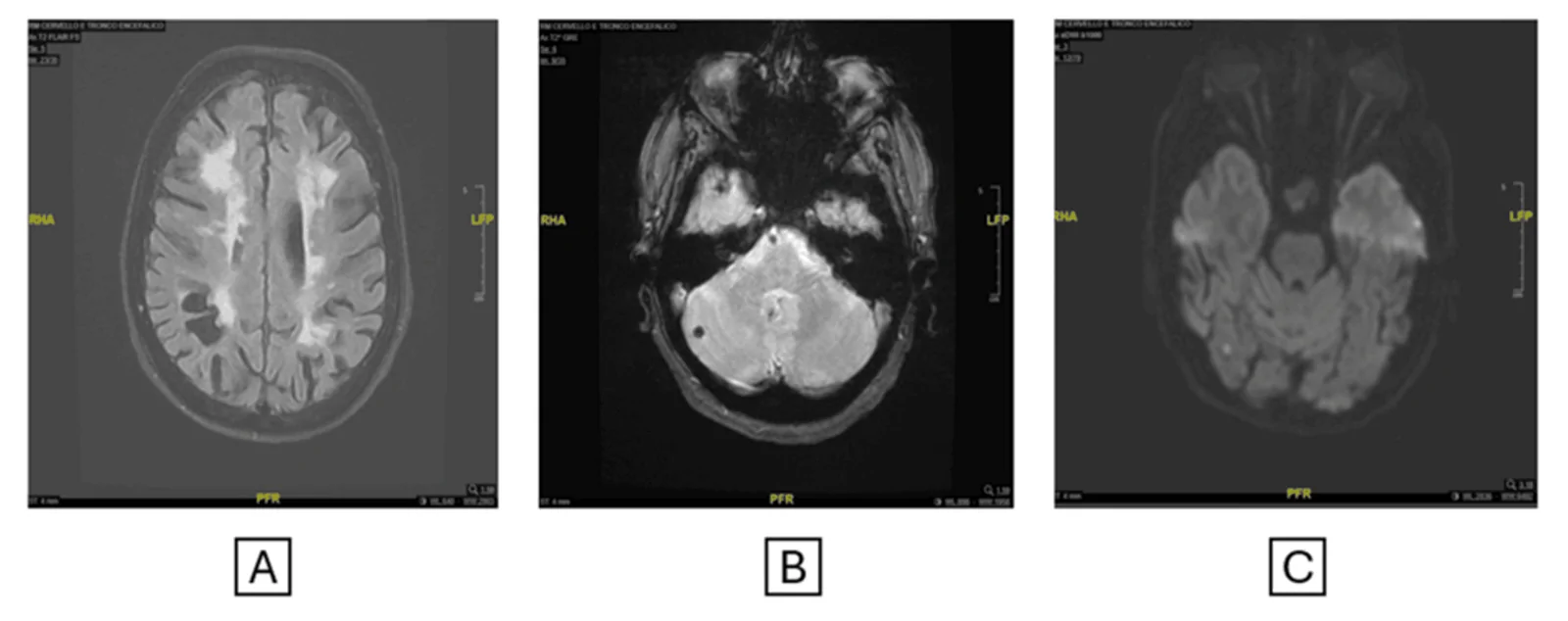
Brain MRI showing multifocal ischemic infarcts. (A) Axial fluid-attenuated inversion recovery (FLAIR) image demonstrating marked chronic ischemic white matter changes. (B) Axial T2-weighted image demonstrating prior hemorrhagic changes. (C) Diffusion-weighted imaging (DWI) demonstrating multiple acute infarcts involving the right frontal subcortical region, vertex, left posterior parietal lobe, and right occipital lobe.

During the current admission, brain MRI demonstrated multiple acute ischemic lesions involving the right frontal subcortical region, vertex, left posterior parietal lobe, and right occipital lobe, superimposed on extensive chronic periventricular white matter ischemic changes with multifocal periventricular gliosis (Figure 1). Repeat cardiological evaluation, including assessment for potential cardioembolic sources, was unremarkable. Vascular imaging demonstrated bilateral carotid atherosclerotic disease without hemodynamically significant stenosis, which did not fully account for the multifocal distribution of cerebral ischemic lesions.

Laboratory investigations revealed mild thrombocytopenia (platelet count 146 × 10³/µL), prolonged activated partial thromboplastin time (aPTT 41.1 seconds), positive lupus anticoagulant (LA) (screening ratio 1.7; confirmatory dilute Russell viper venom time [dRVVT] ratio 1.49), and markedly elevated anti-β2-glycoprotein I (β2GPI) IgG antibodies (1717 CU; reference <20 CU) (Table 1). Anticardiolipin (aCL) IgG and IgM antibodies were negative (8.71 U/mL and 4.33 U/mL, respectively; normal <12 U/mL), indicating that the patient did not have a triple-positive aPL profile.

**Table 1:** Summary of laboratory findings on admission CRP: C-reactive protein; WBC: white blood cell count; MCV: mean corpuscular volume; PT: prothrombin time; INR: international normalized ratio; aPTT: activated partial thromboplastin time; LA: lupus anticoagulant; dRVVT: dilute Russell viper venom time; aCL: anticardiolipin antibodies; β2GPI IgG: beta-2 glycoprotein I immunoglobulin G; LDH: lactate dehydrogenase; ProBNP: pro–B-type natriuretic peptide.

Laboratory Test	Unit	Patient Value	Reference Range
Inflammatory markers			
CRP	mg/dL	0.16	<10
Fibrinogen	mg/dL	474	200–400
Complete blood count			
WBC	×10³/µL	11.41	4.0–11.0
Hemoglobin	g/dL	14.69	12.0–16.0 (F), 13.5–17.5 (M)
MCV	fL	79.7	80–100
Platelets	×10³/µL	146	150–450
Absolute neutrophil count	×10³/µL	8.68	1.5–8.0
Coagulation and antiphospholipid antibody testing			
PT	sec	10.4	11–13.5
INR	—	0.95	0.8–1.2
aPTT	sec	41.1	24–35
Lupus anticoagulant screening	ratio	1.7	<1.2
Lupus anticoagulant confirmation (dRVVT ratio)	ratio	1.49	<1.2
Anticardiolipin IgG	U/mL	8.71	<12 (normal)
Anticardiolipin IgM	U/mL	4.33	<12 (normal)
β2-glycoprotein I IgG	CU	1717	<20
Renal and organ involvement markers			
Urea	mg/dL	65	1–24
Creatinine	mg/dL	2.21	0.7–1.3
Urine protein	mg/dL	600	0–10
LDH	IU/L	458	140–280
ProBNP	pg/mL	438.5	100–400
Metabolic and protein studies			
Glucose	mg/dL	107	80–100
Total protein	g/dL	7.9	6.4–8.3
Albumin	g/dL	4.1	3.6–5.5
Alpha-1 globulin	g/dL	0.4	0.2–0.4
Alpha-2 globulin	g/dL	1.1	0.5–1.0
Beta-1 globulin	g/dL	0.5	0.3–0.6
Beta-2 globulin	g/dL	0.6	0.2–0.5
Gamma globulin	g/dL	1.2	0.7–1.6

According to the Sydney classification criteria, laboratory confirmation of APS requires persistent positivity of LA, aCL antibodies, or anti-β2GPI antibodies on repeat testing performed at least 12 weeks apart. Repeat antiphospholipid antibody testing after 12 weeks confirmed persistent positivity of LA and anti-β2GPI IgG antibodies, confirming APS.

The case was initially discussed at a multidisciplinary meeting involving vascular neurologists and rheumatologists. During the initial admission, long-term anticoagulation was deferred because confirmation of persistent aPL positivity was required according to APS classification criteria. This decision was further influenced by the patient’s increased bleeding risk due to her previous intraventricular hemorrhage and history of intracranial aneurysm repair. Aspirin 100 mg daily was continued, and the patient was transferred to the Rehabilitation Unit for neuromotor rehabilitation.

At rheumatology follow-up, repeat testing confirmed persistent positivity for LA and anti-β2GPI IgG antibodies. Given the confirmed diagnosis of APS and the history of recurrent arterial thrombotic events, warfarin anticoagulation was initiated with a target international normalized ratio (INR) of 2.0-3.0. Low-dose aspirin (100 mg daily) was continued because of concomitant bilateral carotid atherosclerotic disease. At the 90-day post-stroke follow-up, following completion of rehabilitation, the patient achieved complete neurological recovery, with no recurrent ischemic events or intracranial hemorrhage during antithrombotic therapy.

## Discussion

APS typically presents in women of childbearing age; consequently, elderly-onset APS may be overlooked. However, recurrent ischemic strokes in older adults should prompt consideration of APS, as cerebral arterial thrombosis is among the most frequent neurological manifestations of the disease. Elderly patients with APS have been reported to have a higher frequency of lupus anticoagulant (LA) positivity, triple-positive antiphospholipid antibody (aPL) profiles, and arterial thrombotic manifestations [5]. Diagnosing APS in older adults is particularly challenging because ischemic strokes are commonly attributed to competing cardiovascular risk factors, including hypertension, dyslipidemia, and atherosclerotic disease [5,6]. In our patient, her age and history of vascular risk factors initially provided plausible alternative explanations for recurrent cerebrovascular events, contributing to delayed recognition of APS. Furthermore, the absence of clinical features suggestive of systemic autoimmune disease further complicated the diagnostic process.

The differential diagnosis of recurrent ischemic stroke in older adults is broad. It requires careful exclusion of common and potentially treatable causes, including cardioembolism, carotid artery stenosis, malignancy-associated thrombosis, coagulopathies, vasculitis, and embolic stroke of undetermined significance (ESUS) [7]. In our patient, these potential causes were evaluated through comprehensive clinical assessment, echocardiography, computed tomography angiography (CTA), cerebral angiography, brain MRI, and inflammatory marker testing. Imaging demonstrated acute ischemic lesions involving multiple vascular territories without an identifiable cardioembolic source or significant large-vessel stenosis. Additionally, recurrent ischemic events despite appropriate antiplatelet therapy increased suspicion for an alternative prothrombotic mechanism, leading to further evaluation for APS.

Following the Trial of ORG 10172 in Acute Stroke Treatment (TOAST) classification, our patient underwent extensive evaluation, including neuroimaging, vascular imaging, electrocardiography (ECG), and prolonged cardiac rhythm monitoring, to exclude cardioembolic sources and large-artery occlusive disease [8]. Once common etiologies were excluded, further investigation for less frequent causes of recurrent stroke, including hypercoagulable states and autoimmune disorders, was undertaken.

The diagnosis of APS requires the presence of a clinical criterion, such as arterial or venous thrombosis or pregnancy morbidity, together with persistent laboratory evidence of aPL antibodies confirmed on at least two occasions separated by a minimum of 12 weeks [9]. During the diagnostic process, several factors should be considered, including the relationship between clinical manifestations and radiological findings, the contribution of additional thrombotic risk factors, and the specific aPL antibody profile.

Among the different aPL antibodies, LA has the strongest association with thrombotic risk, while triple-positive aPL status identifies patients with the highest risk of recurrent thrombotic events [9,10]. In our patient, aCL IgG and IgM antibodies were negative, indicating that she did not have a triple-positive aPL profile. However, the presence of LA and markedly elevated anti-β2GPI IgG antibodies, together with recurrent arterial thrombotic events involving multiple cerebral vascular territories, represented a clinically significant APS phenotype requiring long-term anticoagulation. Although non-triple-positive patients generally have a lower recurrence risk than those with triple positivity, the thrombotic risk in APS is influenced by the antibody subtype, antibody titers, and clinical manifestations.

Management of APS in patients with multiple comorbidities remains challenging and requires individualized assessment of thrombotic and bleeding risks. In our patient, initiation of anticoagulation required careful consideration because of her history of intraventricular hemorrhage and previous intracranial aneurysm repair. Although this history raised concern regarding hemorrhagic complications, the previous hemorrhagic event was related to a structural vascular lesion that had subsequently been treated, reducing concern for recurrence from the same mechanism. Following confirmation of persistent aPL positivity and multidisciplinary discussion involving vascular neurology, rheumatology, neurosurgery, and hematology, warfarin anticoagulation was initiated for secondary stroke prevention.

Vitamin K antagonists remain the preferred anticoagulant therapy for patients with APS, particularly those with arterial thrombosis and patients with high-risk aPL profiles, including triple-positive APS [11]. Although triple-positive APS is associated with the highest risk of recurrent thrombosis and poorer outcomes, anticoagulation decisions in our patient were primarily guided by her history of recurrent arterial thrombosis, persistent LA positivity, markedly elevated anti-β2GPI IgG antibodies, and overall clinical risk profile. Aspirin was continued because of concomitant bilateral carotid atherosclerotic disease; however, this decision required careful consideration given the increased bleeding risk. Although escalation to a higher INR target or addition of antiplatelet therapy may be considered in selected patients with recurrent arterial thrombosis, our patient was maintained on a standard INR target of 2-3 because of her elevated hemorrhagic risk [11].

Regular clinical follow-up was essential to maintain therapeutic anticoagulation while minimizing bleeding complications. This case highlights the complexity of balancing thrombotic and hemorrhagic risks in elderly patients with APS and multiple cerebrovascular comorbidities and underscores the importance of individualized, multidisciplinary management.

## Conclusions

APS is an autoimmune prothrombotic disorder that may present with recurrent ischemic stroke, including in older adults without previous autoimmune manifestations. This case highlights the diagnostic challenges of elderly-onset APS, where recurrent cerebrovascular events may initially be attributed to conventional vascular risk factors. In this patient, brain MRI demonstrated multifocal acute ischemic infarcts involving multiple vascular territories, with extensive chronic ischemic changes and previous hemorrhagic changes, reflecting the complexity of her cerebrovascular disease. The diagnosis of APS was established through recurrent arterial thrombotic events and persistent positivity for LA and anti-β2GPI IgG antibodies, despite the absence of triple-positive aPL status. Management required careful balancing of recurrent thrombotic risk against hemorrhagic complications, particularly given the history of intracranial hemorrhage and aneurysm repair. This case emphasizes the importance of considering APS in older patients with recurrent ischemic stroke when conventional etiologies do not fully explain the clinical picture and highlights the need for individualized, multidisciplinary decision-making regarding anticoagulation.
